# Wounding promotes ovarian cancer progression and decreases efficacy of cisplatin in a syngeneic mouse model

**DOI:** 10.1186/s13048-018-0428-6

**Published:** 2018-07-04

**Authors:** Yooyoung Lee, Alexandra Kollara, Taymaa May, Theodore J. Brown

**Affiliations:** 1 Division of Gynecologic Oncology, Princess Margaret Hospital Cancer Centre, Toronto, ON Canada; 2 Lunenfeld-Tanenbaum Research Institute at Sinai Health Systems, Mt. Sinai Hospital, 60 Murray Street, 6-10016-3, Toronto, ON M5T 3L9 Canada; 3 Department of Obstetrics and Gynecology, University of Toronto, Toronto, ON Canada

**Keywords:** Ovarian cancer, ID8 cells, Wound healing, Cisplatin, Mouse, Chemoresistance

## Abstract

**Background:**

Primary cytoreductive surgery followed by adjuvant chemotherapy is the standard treatment for advanced epithelial ovarian cancer. The average interval between surgery and chemotherapy initiation is approximately 4-weeks at most centers; however, since surgery may accelerate residual tumor growth, a shorter interval may be more beneficial.

**Methods:**

The murine ID8 cell model of ovarian cancer was used to examine the efficacy of cisplatin treatment administered perioperatively or 7 days after surgical wounding. Luciferase-expressing cells ID8 cells were injected intraperitoneally (i.p.) into female C57/Bl6 mice. Fourteen days post-injection, animals received an abdominal incision or anesthesia alone and received i.p. cisplatin either on the surgical day or 7 days later, or received no chemotherapy. Additional animals received cisplatin 28 days after wounding for comparison.

**Results:**

Abdominal tumor mass increased 2.5-fold in wounded vs. unwounded animals as determined by bioluminescent in vivo tumor imaging. Cisplatin administered on the day of wounding decreased tumor burden by 50%, as compared to 90% in unwounded animals. Cisplatin on day 7 or day 28 decreased tumor burden by 80 and 37% respectively.

**Conclusions:**

Surgical wounding increases ovarian tumor mass and decreases perioperative cisplatin efficacy in this animal model. Administration of cisplatin 1 week after surgery was more effective than cisplatin administered perioperatively or 4 weeks after surgery.

**Electronic supplementary material:**

The online version of this article (10.1186/s13048-018-0428-6) contains supplementary material, which is available to authorized users.

## Background

Advanced epithelial ovarian cancer has a poor prognosis with a 5-year survival rate of less than 40% [1]. Standard treatment consists of a combination of maximal cytoreductive surgery followed by adjuvant chemotherapy to eliminate residual macroscopic or microscopic disease [2, 3]. Despite this approach, disease recurs in more than 70% of patients, underscoring the inability of this treatment strategy to cure the disease. Ovarian cancer patients who achieve microscopic as compared to macroscopic residual disease at primary surgical cytoreduction have a significantly longer overall survival [4]. Thus, a key therapeutic goal is to optimize chemotherapy efficacy to maximize residual tumor elimination.

Ovarian cancer patients generally begin adjuvant chemotherapy 3–5 weeks after primary cytoreductive surgery [5], although there is no consensus regarding the optimal postoperative waiting period. Delayed initiation of chemotherapy could negatively impact survival since factors released during wound healing can act to promote proliferation of residual tumor cells [6, 7]. A case series of 8 patients with testicular cancer found dramatic exacerbation of residual disease after cytoreductive surgery [8]. Similarly, work by Retsky and colleagues [9] indicated that surgery may reawaken dormant breast cancer cells. A tumor-promoting effect of surgery is further supported by studies using animal models of breast and colorectal cancers, where incomplete or non-curative surgery was performed to determine the impact on residual disease. In these models, surgery increased proliferation of residual tumor cells and accelerated tumor growth [10, 11].

Administration of chemotherapy near the time of surgery may mitigate surgery-induced increased tumor growth since actively dividing cells are most susceptible to chemotherapeutic agents. Reducing the interval between surgery and initiation of adjuvant chemotherapy associates with better survival in patients with early breast [12] and colorectal cancer [13]. In a post-trial ad hoc analysis of Gynecologic Oncology protocol 218, which investigated the time from surgery to initiation of chemotherapy in advanced ovarian carcinoma, Tewari et al. [5] found earlier initiation of chemotherapy (time intervals less than 25 days) was associated with improved overall survival in those patients with microscopic residual disease. Importantly, a study with cyclophosphamide in a subcutaneous mouse mammary tumor model indicates that perioperative administration of chemotherapy is most effective in decreasing residual tumor growth after cytoreductive surgery [14]. Surprisingly, studies addressing the interval from primary surgery to adjuvant chemotherapy in animal models of ovarian cancer have not been reported.

In the present study, we used the widely accepted ID8 animal model of ovarian cancer to determine if surgical abdominal wounding impacts peritoneal dissemination of tumor expansion and the efficacy of cisplatin administered at either the time of surgical wounding or 1 week later. These cells were derived by Roby and coworkers [15] from mouse ovarian surface epithelial cells that had spontaneously transformed in repeated passage tissue culture. Advantages to this model include its recapitulation of high-grade serous ovarian cancer [16], the most commonly diagnosed epithelial ovarian cancer histotype, and the ability to use non-immunocompromised syngeneic C57Bl6 mice. In this model, cells were injected intraperitoneally (i.p.) at a level to reflect a clinical situation of microscopic residual disease following cytoreductive surgery.

## Methods

### Cell culture

ID8-luciferase expressing cells were generated by stably co-transfecting ID8 cells (obtained from Dr. Jim Petrik, University of Guelph, Guelph, ON, Canada) with pCMV-hyPBase (Trust Sanger Institute, Cambridge, UK) [17] and the PB-CAG-Luciferase-IRES-eGFP-pA vector (provided by Dr. Andras Nagy, Lunenfeld-Tanenbaum Research Institute, Toronto, ON, Canada), in 1:3 ratio using lipofectamine 2000 (Invitrogen, Burlington, ON, Canada). Cells were grown in RPMI 1640 medium without phenol red, supplemented with 5% heat-inactivated normal fetal bovine serum, 100 units/mL penicillin and 100 μg/mL streptomycin (Invitrogen) in a humidified chamber with 5% CO_2_ at 37°C. Transfected cells were clonally selected in medium supplemented with 1 μg/ml puromycin (Invitrogen) and were initially screened for luciferase activity using a Luciferase Assay System (Promega) and a GloMAX microplate luminometer (Promega, Madison, WI, USA).

### Animals

C57/Bl6 female mice 6–8 weeks of age were obtained from Charles River Laboratories (Sherbrooke, QC, Canada) and group-housed under standard conditions in accordance with the guidelines of the Canadian Council on Animal Care. Mice were maintained on a 12–12 h light-dark schedule and were provided food and water ad libitum. All animal procedures were approved by the University of Toronto Animal Care and Use Committee.

ID8-Luc 11 cells (5 × 10^6^ cells in 0.2 ml phosphate-buffered saline; PBS) were injected intraperitoneally (i.p.) to mimic peritoneal dissemination. Surgical wounding simulating a laparotomy was performed under isoflurane anesthesia (Baxter, Deerfield, IL, USA) and consisted of a single 1.5 cm midventral abdominal incision through the skin and musculature and gentle exploration of the intra-peritoneal cavity. The abdominal wall was closed with absorbable sutures. Control animals were subjected to anesthesia without surgical wounding. Cisplatin (Sigma, St. Louis, MO, USA) was dissolved in PBS (1.4 mg/ml) and administered i.p. as a single dose (14 mg/kg) [18] immediately after surgery, or 7 or 28 days after surgery. Animals were evaluated daily for tumor growth, ascites accumulation, and postoperative complications including wound problems. Animals were weighed and given health assessments at least every other day. Animals were euthanized by CO_2_ inhalation followed by cervical dislocation 12 weeks after tumor cell inoculation or upon evidence of excessive ascites formation, 20% weight gain or loss, signs of debilitation, or wound ulceration.

### In vivo bioluminescent imaging

Bioluminescent imaging was performed on an IVIS Spectrum In Vivo Imaging System (Perkin Elmer, Rodgau, Germany). As recommended by the manufacturer, the auto-exposure setting was used to automatically set the exposure time, f/stop and binning to keep the signal within an optimal range for quantification and to avoid overexposure during image acquisition. Auto-exposure sensitivity settings used for the snapshot image were adjusted to obtain a minimal target count of 3000. Luminescence was measured as total flux (photons per second (P/S)).

Two highly expressing clones (ID8–11 and ID8–15) were subjected to bioluminescent imaging to select the best clone for in vivo study. Cells were seeded in 100 μl medium at varying concentrations into 96-well black culture plates. An equal amount of in vivo glow solution (D-Luciferin, Promega) was added just prior to imaging. Images were acquired with a 12.8 cm width field of view, 2 × 2 binning factor, and an exposure time of 1 s.

For in vivo imaging of dissemination of tumor cells, animals were anesthetized with 1.5–2.5% isoflurane inhalation, maintained throughout the imaging procedure. Animals received 150 mg D-luciferin/kg body weight i.p. at a concentration of 15 mg/L, 8 min prior to imaging. Images were acquired with a 12.8 cm field of view, 4 × 4 binning factor, and an exposure time ranging from 0.5–1 s.

### Serum collection

Blood was collected via cardiac puncture just prior to necropsy and centrifuged for 10 min at 500 g. Serum from each group of animals were pooled and filtered through a 0.22-μm syringe filter (Millipore, Burlington, MA, USA) and stored at − 80 °C.

### XTT dye-reduction assay

ID8 or ID8 luciferase-expressing cells (ID8–11 and ID8–15) were seeded into 96-well plates at 2.0 × 10^3^ cells per well. Cisplatin was added 24 h after seeding and cell number was determined by XTT dye-reduction assay. Briefly, 50 μl of XTT (Invitrogen) solution (1 mg/ml) were added to each well. Following incubation for 3 h in a humidified 5% CO_2_ atmosphere at 37 °C, reduced XTT was measured spectrophotometrically at 492 nm using a microtiter plate reader.

To determine the impact of serum on cell growth, ID8 cells were seeded into 96-well plates at 2.0 × 10^3^ cells per well. Culture medium was replaced 24 h after seeding with fresh medium supplemented to 10% (vol/vol) with mouse serum. Cells were incubated in the presence or absence of 10 μM cisplatin for 72 h. Cell number was determined by XTT dye reduction as described.

### Statistical analysis

Data are expressed as mean ± SEM and were analyzed by one-way ANOVA followed by a Fisher LSD Multiple Comparison Test using Prism v7 (Graphpad Software, La Jolla, CA, USA). A natural log transformation was applied to the datasets if Bartlett’s test indicated unequal variances. Survival outcome is presented as Kaplan-Meier survival curves and was analyzed using the Log-Rank (Mantel-Cox) Test. Data on ascites development were compared using a Chi-square analysis. Data were considered statistically significant at *p* < 0.05.

## Results

**Fig. 1:**
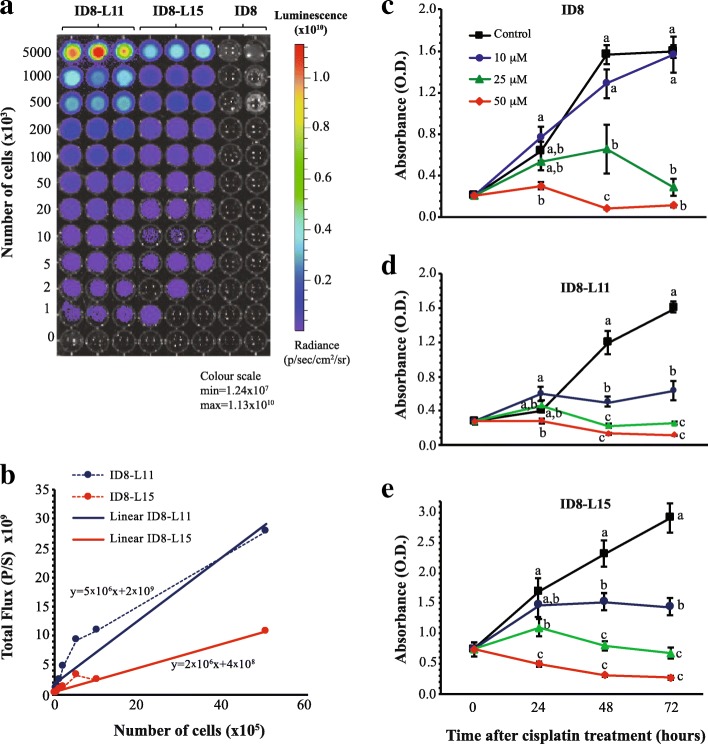
Bioluminescent luciferase activity and sensitivity to cisplatin of ID8-L11 and ID8-L15 cells in vitro*.* **a** Concentration-dependent bioluminesence imaging of both cell sublines compared to non-luciferase expressing parental ID8 cells. Different concentrations of cells were added to a 96-well tissue culture plate and imaged on an IVIS Spectrum In Vivo Imaging System. **b** Graphical representation of the quantitation of images shown in Panel A (dashed lines). Solid lines indicate a linear fitting of the data. **c**-**e** Response of cells to cisplatin. Parental ID8 (**c**), ID8-L11 (**d**), or ID8-L15 (**e**) cells were seeded into 96 well plates. One day later, cells were treated with 0, 10, 25, or 50 μM cisplatin and relative cell viability was determined by a XTT dye-reduction assay. Points represent the mean ± SEM of 8 determinations. Within each time point, points with different letters are statistically different from one another as determined by ANOVA followed by SNK multiple comparison test (*p* < 0.05)

ID8 cells were transfected with a constitutively active luciferase expression vector to enable noninvasive longitudinal assessment of tumor burden in vivo. Transfected cells were clonally selected and screened for luciferase activity. Out of 15 clones screened, ID8-L11 and ID8-L15, were found to express high levels of luciferase activity (Additional file 1: Table S1) and robust growth. Luciferase activity assays and bioluminescent imaging of these two sublines in vitro indicated a linear relationship between luciferase activity and cell number (Fig. 1a and b). Parental ID8 cells were growth inhibited in culture by 25 or 50 μM cisplatin (Fig. 1c), with both cell sublines exhibiting similar sensitivity to these doses and to 10 μM cisplatin (Fig. 1d and e). ID8-L11 cells produced a stronger bioluminescence signal than ID8-L15 cells, imparting a greater sensitivity for detection while maintaining a dose-dependent response to cisplatin; therefore, these cells were selected for further study.

**Fig. 2:**
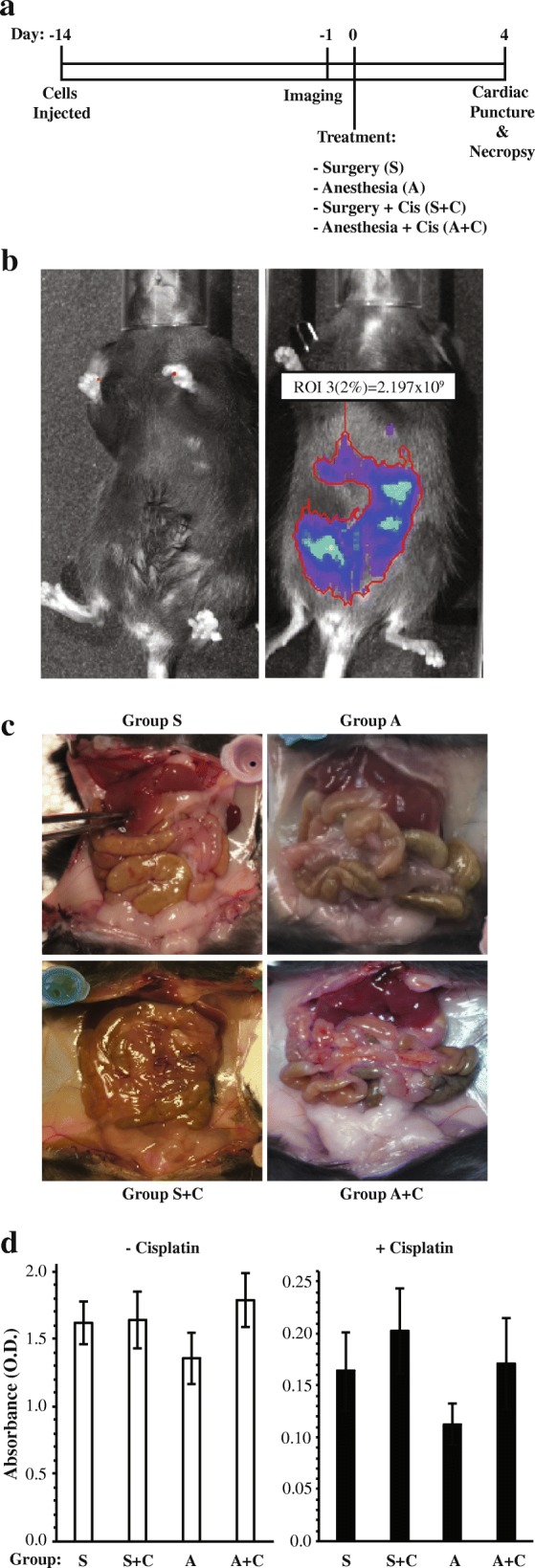
In vivo imaging of ID8-L11 cells and effect of serum from wounded mice on growth of parental ID8 cells. **a** Schematic of the treatment paradigm used in the preliminary study. **b** Representative bioluminescence images of a mouse not injected with ID8-L11 cells (left) and a mouse injected with 5 million ID8-L11 cells i.p. 13 days earlier (right). **c** Representative images of the abdominal viscera of mice in each of the four treatment groups at time of necropsy. No evidence of macroscopic seeding was found. **d** Serum from mice of the different treatment groups did not impact the growth of parental ID8 cells in vitro. Cells were grown in medium supplemented to 10% with serum pooled from within the four treatment groups in the presence or absence of 25 μM cisplatin. XTT dye reduction assay was performed 72 h after initiation of treatment. Bars represent the mean ± SEM of 8 replicates. Two-way ANOVA performed on natural log-transformed data indicated a statistically significant effect of cisplatin but no statistically significant effect of serum from the different treatment groups or interaction

The ability to detect ID8-L11 cells in vivo was tested in a preliminary study. C57/Bl6 mice were injected i.p. with 5 × 10^6^ cells 13 days prior to imaging. On day 14, mice were anesthetized and subjected to surgical wounding (abdominal incision; *n* = 6) or left unwounded (*n* = 6). Half of the animals in each group were treated with i.p. cisplatin and animals were sacrificed 4 days later following cardiac puncture (Fig. 2a). Bioluminescence was not detectable in control mice not injected with ID8-L11 cells (*n* = 3) whereas all mice injected with the cells exhibited abdominal luminescence consistent with the presence of luciferase-expressing cells (Fig. 2b). Neither macroscopic tumor formation nor ascites were detected at the time of necropsy in any of the animals (Fig. 2c), indicating that this model is representative of microscopic residual disease following primary cytoreductive surgery.

To determine if factors released into the serum as a result of wounding affect the growth of cancer cells in vitro, ID8 cells were cultured with medium supplemented to 10% with pooled sera obtained from the four treatment groups, in either the presence or absence of 25 μM cisplatin. No difference in cell viability or response to cisplatin was apparent due to exposure to sera from the different treatment groups (Fig. 2d; *p* > 0.05).

**Fig. 3:**
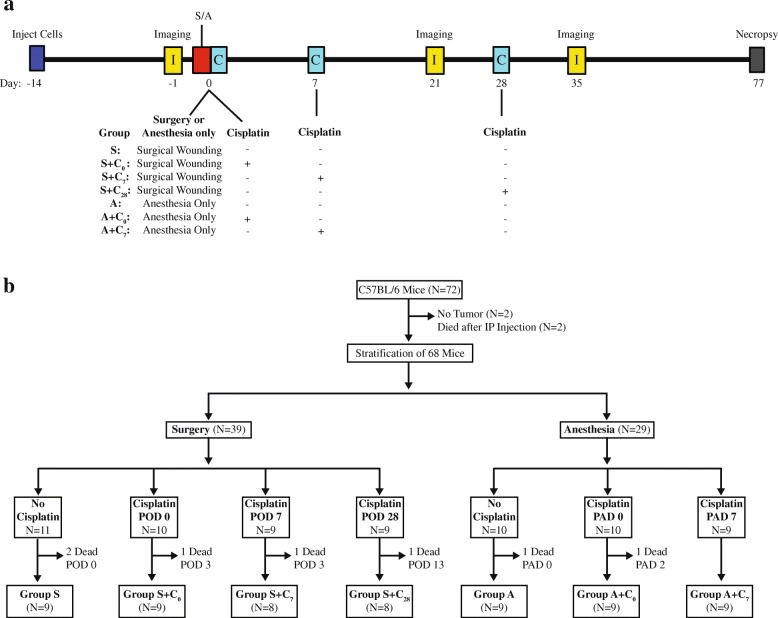
Study treatment paradigm. **a** Schematic showing the overall treatment and imaging schedule used in the study. **b** Schematic showing the stratification of imaged animals to the seven treatment groups and the loss of animals within groups. S = surgery, A = anesthesia, C_0_ = cisplatin on the perioperative day, C_7_ = cisplatin on postoperative day (POD) 7, C_28_ = cisplatin on POD 28. All animals were inoculated i.p. with 5 million ID8-L11 cells and were imaged 13 days later

To determine the impact of surgical wounding on disease progression and sensitivity to cisplatin, 72 mice were injected with ID8-L11 cells and imaged 13 days later. Two mice died prior to imaging and two mice had no detectable tumor cells. The remaining animals were stratified to the amount of disease present in pretreatment imaging and were assigned to seven treatment groups: Group S = surgery alone; Group S + C_0_ = surgery plus cisplatin on the same day; Group S + C_7_ = surgery plus cisplatin on post-operative day (POD) 7; Group S + C_28_ = surgery plus cisplatin on POD 28; Group A = anesthesia alone; Group A + C_0_ = anesthesia plus cisplatin on the same day; Group A + C_7_ = surgery plus cisplatin on POD 7. Animals were reimaged 21 or 35 days after wounding or control (anesthesia only) treatment (Fig. 3a). Several mice died during or within 3 days of surgery/anesthesia or were euthanized because of superficial wound dehiscence (Fig. 3b). As a result, 61 mice were imaged at all three time points and were further followed for indications of significant ascites (moribund), excessive weight gain or loss, or debilitation (cachexia, hunched back with tremor, increased respiratory frequency), which were taken as a surrogate for death. The study was terminated 13 weeks after tumor cell injection.

**Fig. 4:**
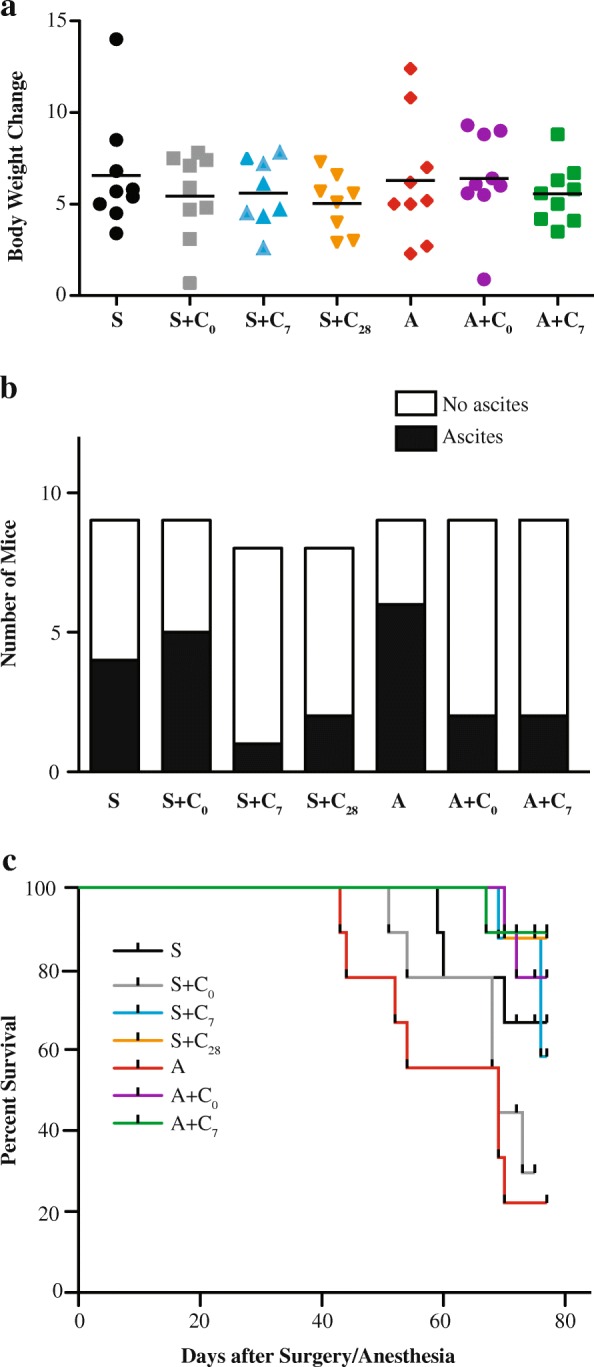
Change in body weight, incidence of ascites (Panel **b**) and survival curves for the seven treatment groups. **a** Change in body weight from the time of cell injection to animal sacrifice is shown for each treatment group. Lines represent group mean. No changes were detected using ANOVA. **b** Numbers of mice that exhibited signs of ascites development within the treatment groups. No overall effect due to treatment group was detected using a Chi-square analysis. **c** Kaplan-Meier growth curves obtained for each of the treatment groups using indicators of animal health/distress as a surrogate for survival. An overall effect of treatment was indicated by a Log-Rank test (*p* = 0.0012)

There were no differences between groups in overall change in body weight during the study duration (Fig. 4a, *p* = 0.8271). Animals assigned to anesthesia alone appeared to have the greatest incidence of ascites development (Fig. 4b); however, chi-square analysis indicated the differences in ascites occurrences between treatment groups was not statistically significant (*p* = 0.160).

There was a statistically significant overall impact of treatment group on survival (*p* = 0.0012). In general, lower survival was observed for mice that received anesthesia alone or surgery combined with perioperative cisplatin. Highest survival was observed for animals that received cisplatin on day 7, regardless of surgical wounding, or in non-wounded animals that received cisplatin on day 0 (Fig. 4c). Multiple comparison testing using a Bonferroni-corrected statistical threshold indicated that cisplatin treatment resulted in improved survival in non-wounded animals but not in wounded animals.

**Fig. 5:**
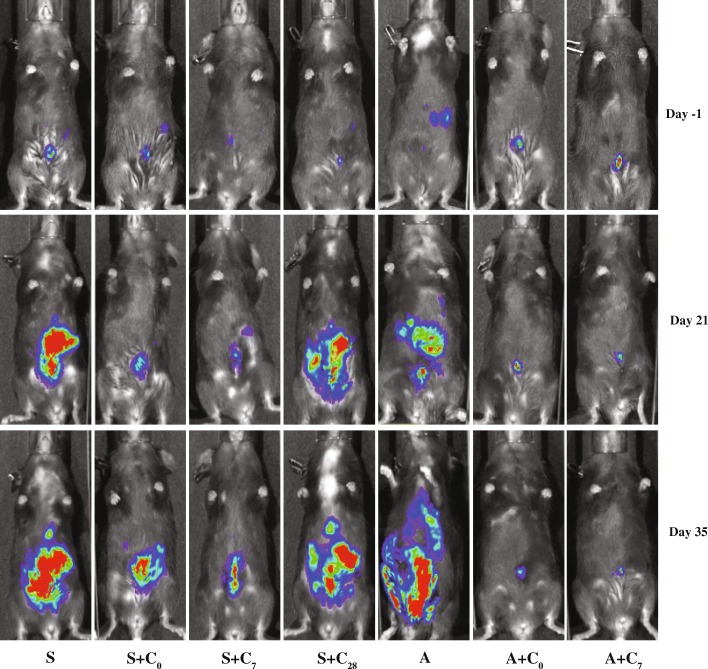
Representative bioluminescence images obtained for the seven treatment groups at each of the three imaging sessions

**Fig. 6:**
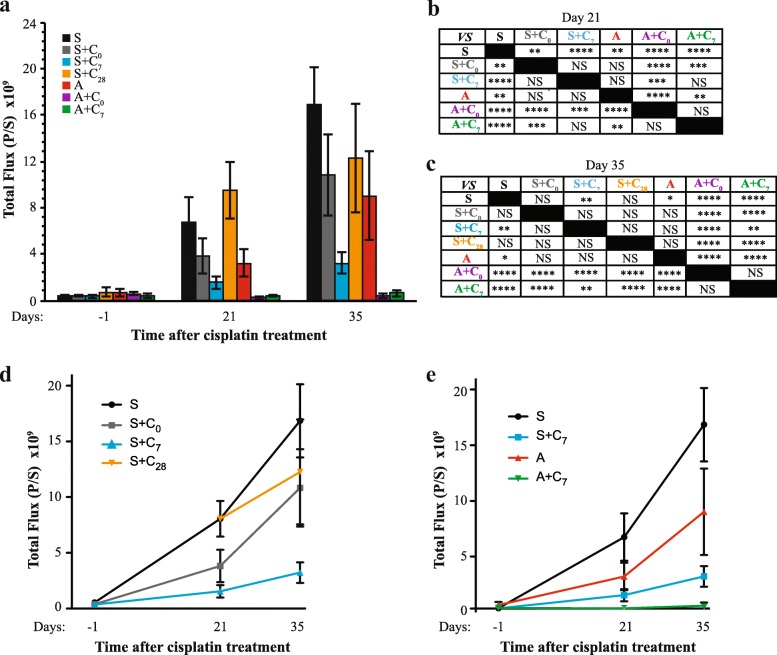
Compiled results from the imaging of animals at day − 1, 21, and 35 relative to the day of surgical wounding. **a** Mean levels of abdominal tumor burden measured for each of the seven treatment groups. Bars represent the mean ± SEM. **b** Statistical results obtained for imaging of animals on day 21. For this analysis, mice assigned to treatment groups S and S + C_28_ were combined. Data were subjected to a natural log transformation prior to analysis by ANOVA followed by Fishers LSD multiple comparison test. *p < 0.05, **p < 0.01, ***p < 0.001, ****p < 0.0001, NS = not significant. **c** Statistical results obtained for imaging of animals on day 35. Data were subjected to a natural log transformation prior to analysis by ANOVA followed by Fishers LSD multiple comparison test. **d** and **e** Separation of the data shown in (panel **a**) to highlight the impact of cisplatin in surgically wounded animals (Panel **d**) and comparing the effect of surgery and cisplatin administered on day 7 to sham-wounded animals (Panel **e**). Points represent the mean ± SEM

Bioluminesence imaging was performed on day 21 and 35 after wounding to assess the impact of treatment on tumor burden. Representative images are shown in Fig. 5. Quantitation of abdominal bioluminescence determined on day 21 indicated that animals subjected to wounding had the greatest tumor burden, with 2.5-fold higher levels of tumor cells found in animals that had not received cisplatin compared with animals subjected to anesthesia only (Fig. 6). Cisplatin treatment administered on either the perioperative day or on day 7 reduced tumor burden in both wounded and non-wounded animals, with lower levels achieved in non-wounded animals. Cisplatin administered on the same day as wounding decreased the collective peritoneal tumor mass by approximately 50%, which was 18-fold more than the tumor mass measured in non-wounded animals treated with cisplatin at the same time. Cisplatin treatment in non-wounded animals reduced tumor burden by more than 90%. Administration of cisplatin 1 week after wounding was more effective, resulting in a reduction of tumor mass by approximately 80%, which was not statistically different from the tumor mass measured in non-wounded animals treated with cisplatin at the same time. Cisplatin treatment in non-wounded animals at this time reduced tumor burden by a similar percentage, approximately 89%. Importantly, the levels of bioluminescence measured in wounded animals treated with cisplatin on either day were not statistically different from levels measured in non-wounded animals that had not received cisplatin, indicating that perioperative cisplatin mitigated the tumor promoting effects of wounding.

While the primary objective of the study was to compare perioperative cisplatin to cisplatin administered on day 7 following wounding, an additional group of wounded animals treated with cisplatin on day 28 was included as a comparator to reflect the current average waiting period to initiate adjuvant chemotherapy [19]. As determined by imaging performed 1 week later (day 35), wounded mice receiving chemotherapy on day 28 had 73% of the amount of tumor mass as untreated wounded mice (Fig. 6). This decrease was not statistically significant. At this imaging time point, wounded animals not treated with cisplatin showed highest levels of tumor burden; however, this level was not statistically different from that measured in untreated non-wounded animals. Of wounded animals, those treated with cisplatin on day 7, but not on the perioperative day, exhibited reduced tumor burden compared to untreated wounded animals. In contrast, cisplatin administered to non-wounded animals during the perioperative period or on day 7 effectively reduced tumor burden.

## Discussion

Using the ID8 syngeneic ovarian cancer mouse model, we report that surgical wounding increased growth of tumor cells within the peritoneal cavity, as determined by quantitation of bioluminescence 3 weeks later. While cisplatin decreased tumor burden, its efficacy was affected by surgical wounding and timing of chemotherapy. Cisplatin administered on the day as wounding decreased collective peritoneal tumor mass by only 50%, whereas more than a 90% reduction was measured in non-wounded animals. Administration of cisplatin 1 week after wounding appeared more effective, resulting in a reduction of tumor mass by 80%. These results indicate that surgical wounding negatively impacts the efficacy of perioperatively delivered cisplatin, suggesting that systemic factors released at the time of wounding or during wound healing protect cells from cisplatin-induced cell death.

As determined by imaging performed on day 35, chemotherapy on day 28 reduced tumor burden by 27%, which did not differ statistically from the level of tumor cells measured in wounded animals that had not received chemotherapy. In contrast, wounded mice treated with cisplatin perioperatively or on day 7 had 65 and 19%, respectively, of the tumor mass measured in untreated wounded animals at this time point. Our results thus support the assertion that earlier administration of cisplatin following surgery is more efficacious.

Studies have indicated that surgical wounding promotes the growth and metastasis of solid tumors [20, 21]. Ramolu et al. [22] found that fluid taken from wounds 24 or 48 h after breast surgery stimulated the growth of MCF-7 and HCC1937 cancer cells, but not non-malignant MCF-10A cells, in vitro. While this and a similar study [23] have been taken as evidence to explain recurrence at the operative site, other studies have shown that serum taken from wounded patients similarly stimulates growth of cancer cell lines [24], indicating that systemic factors released during wounding or wound healing may promote tumor progression. Healing of acute wounding consists of 4 phases: coagulation and hemostasis (immediate after injury), inflammation (24–72 h), cell proliferation (72 h to 2 weeks), and tissue remodeling [25]. Thus, in our model, factors that stimulate a more rapid expansion of tumor cells may be restricted to the abdominal cavity or may be present during a specific phase of wound healing. We did not detect differences in ID8 cell growth in vitro when culture medium was supplemented with serum from wounded animals or animals treated with cisplatin. However, since we collected serum samples 4 days after wounding, it remains possible that differences might be seen with serum collected nearer the time of wounding.

A key finding of our study, that perioperative cisplatin efficacy was inhibited by wounding, was unexpected. Surgical wounding has been reported to activate quiescent tumor cells [26]. The mitotic activity of residual cancer cells increases within 1 day of surgery [27] and remains elevated for 1–2 weeks [26]. Since actively dividing cells are more susceptible to cisplatin [28], we hypothesized that the perioperative environment would maximize cisplatin effects. Moreover, an animal study performed more than 30 years ago by Fisher et al. [14] with transplanted mouse syngeneic mammary cancer cells indicated that preoperative or perioperative cyclophosphamide treatment was more effective than treatment administered 3 days later. In fact, a delay of treatment for 1 week was least effective.

Aside from the chemotherapy and cell line used, key differences in the design of these two studies could contribute to the discrepancy in findings. The model used by Fisher et al. [14] consisted of subcutaneously injecting tumor cells into both hind legs of mice. Surgery, consisting of amputating the leg inoculated with the greater number of cells (the primary tumor), increased tumor growth in the remaining leg. The macroscopic amount of residual disease remaining at the time of surgery, its subcutaneous location, and the extent of surgery differed from the microscopic i.p. disease and incision-wounding modeled in our study. Interestingly, a recent animal study reported that expression of survivin in residual tumor tissues, taken as a surrogate marker of chemoresistance, began increasing within 6 h of surgery, peaked at post-operative day 2, and decreased to baseline levels by post-operative day 7 [29]. This suggested chemoresistance pattern during the immediate post-operative period is consistent with our finding of impaired cisplatin activity perioperatively vs. 7 days later.

An alternative interpretation of our data is that perioperative cisplatin mitigates the impact of surgical wounding on residual tumor expansion. The level of tumor burden in wounded animals treated with perioperative cisplatin was equivalent to untreated non-wounded animals at both imaging sessions. This has important clinical implications as there is growing interest in the use of hyperthermic intraperitoneal chemotherapy (HIPEC) in ovarian cancer. HIPEC is typically administered intraoperatively at the time of cytoreductive surgery and enables direct administration of a high volume of systemic therapy with a homogenous distribution at the nadir of residual disease. In select patients with colorectal carcinoma and peritoneal carcinomatosis, perioperative i.p. chemotherapy enhanced the overall median survival, particularly for patients in which surgery resulted in microscopic residual disease [30]. Although a recent randomized clinical trial showed no benefit of HIPEC on survival in patients with advanced ovarian cancer [31], they noted a trend favoring HIPEC in patients that had received neoadjuvant chemotherapy. This has since been demonstrated in a recent report by van Driel et al. [32], who showed increased progression-free and overall survival of stage III ovarian cancer patients randomized to receive interval cytoreductive surgery with HIPEC vs. interval cytoreductive surgery alone. Further studies examining the impact of surgical wounding and efficacy of i.p. chemotherapy in a model of neoadjuvant chemotherapy are warranted.

A possible mechanism underlying surgery-driven accelerated residual tumor growth may be an increase in the level of angiogenic cytokines during the perioperative period. For example, perioperative levels of systemic proangiogenic cytokines such as vascular endothelial growth factor (VEGF), basic fibroblast growth factor (bFGF) and transforming growth factor beta (TGF-β) are increased in blood samples drawn from breast, lung, and gastric cancer patients during the perioperative period [33–35]. VEGF levels were also found to be increased in wound fluid collected during the perioperative period [23, 24]. Other factors released during wound healing that have been implicated in promoting tumor growth and metastases include epidermal growth factor, activin, platelet-derived growth factor, and prostaglandin E_2._ Limited studies conducted with inhibitors to many of these growth-promoting factors have indicated partial mitigation of surgery-induced growth; however, it is likely that the response is multifactorial. Indeed, Hofer et al. [23] found that the combination of TGF-β and bFGF better replicated the impact of wound fluid on the growth of melanoma cells in vivo than either growth factor alone. While these studies have focused on surgery-stimulated cell proliferation, studies have not yet addressed potential mechanisms by which wound-associated factors may promote cell survival pathways to diminish the impact of chemotherapy.

In vivo imaging used in this study allowed us to monitor the change of tumor burden non-invasively. A potential concern regarding bioluminescent imaging in the abdominal cavity is that ascites formation at the time of imaging could underestimate the cell number due to dilution of luciferin substrate by excessive ascites [36]. However, none of the mice injected with ID8-L11 cells had obvious ascites during the three imaging sessions and a supra-saturating concentration of substrate was used.

While clinically relevant, there are limitations of our model that caution direct application to humans. As opposed to human high-grade serous ovarian cancer cells, ID8 cells do not bear a p53 mutation and have intact *BRCA1* and *BRCA2* expression [37]. It is possible that p53-mutated cells or cells with diminished functional BRCA1 respond differently. Indeed, HGSOC with diminished BRCA1 or BRCA2 expression typically have increased sensitivity to DNA damaging chemotherapeutics and restoration of BRCA1 in a BRCA1 deficient breast cancer cell line decreased cisplatin sensitivity in a xenograph model [38, 39]. Additionally, we administered only a single dose of cisplatin rather than a full course of treatments as would occur clinically. Also, the wound healing process in humans differs from that of mice. For example, wound healing of many animals, including the mouse, occurs via contraction rather than epithelization as in humans. However, similar to humans, incisional and excisional wounding in mice typically completes within 1–2 weeks post-injury [40], with many similarities in cellular and molecular responses to humans [41]. Lastly, our bioluminescence results showing residual tumor burden did not correlate with survival data. We relied chiefly on behavioral parameters and significant accumulation of ascites as surrogates of survival for humanitarian reasons. Thus, we feel that survival data are less informative in the context of the current study than our bioluminescence data.

One concern regarding early initiation of chemotherapy in clinical practice is wound complications or morbidity. We did not find wound complications or increased morbidity associated with early chemotherapy. These findings agree with a study investigating the effect of early post-operative chemotherapy on wound healing in patients with ovarian cancer [42]. In the study, early chemotherapy did not increase the risk of wound complications despite efforts to begin chemotherapy as soon as possible after cytoreductive surgery and neither the frequency of bowel resection nor type of fascial or skin closure adversely influenced the risk. In addition, early post-operative IP (EPIC) chemotherapy, which usually starts within 1 week after surgery, is not associated with increased morbidity in ovarian cancer [43]. Given the safety regarding early chemotherapy in animal and clinical studies, a clinical trial would be warranted to explore the feasibility and efficacy of perioperative vs. EPIC chemotherapy.

## Conclusion

The results of this study indicate that surgical wounding enhanced peritoneal tumor burden in a syngeneic model of ovarian cancer. While administration of cisplatin at the time of surgical wounding mitigated the effect on tumor progression, the efficacy of this cisplatin appeared to be reduced. While direct relevance to humans is limited since the surgical wounding in this model did not include reducing tumor burden, these findings highlight the need for further studies investigating factors released at the time of surgery that impact tumor cell survival.

## Additional file

Additional file 1: **Table S1.** Luciferase activity levels of clonally selected ID8 cells stably transfected with PB-CAG-Luciferase-IRES-eGFP-pA vector. (DOCX 15 kb)

## Abbreviations

bFGF: Basic fibroblast growth factor
HIPEC: Hyperthermic intraperitoneal chemotherapy
i.p: Intraperitoneally
PBS: Phosphate-buffered saline
POD: Post-operative day
TGF-β: Transforming growth factor beta
VEGF: Vascular endothelial growth factor
