# Supplementary material for: Wounding promotes ovarian cancer progression and decreases efficacy of cisplatin in a syngeneic mouse model
Source: J Ovarian Res. 2018 Jul 4;11:56. doi: 10.1186/s13048-018-0428-6 (PMC6032528; doi:10.1186/s13048-018-0428-6)
Supplement: Supplementary file 1 — Table S1. Luciferase activity levels of clonally selected ID8 cells stably transfected with PB-CAG-Luciferase-IRES-eGFP-pA vector. (DOCX 15 kb) [file 13048_2018_428_MOESM1_ESM.docx]

**Table S1**: Luciferase activity levels of clonally selected ID8 cells stably transfected with PB-CAG-Luciferase-IRES-eGFP-pA vector.

| **Cell ID** | **Absorbance (O.D.)** |
| --- | --- |
| ID8 | 246 |
| ID8-1 | 463 |
| ID8-2 | 2650 |
| ID8-3 | 664 |
| ID8-4 | 3473114 |
| ID8-5 | 1112 |
| ID8-6 | 3082 |
| ID8-7 | 6604 |
| ID8-8 | 250919 |
| ID8-9 | 6841 |
| ID8-10 | 195276 |
| **ID8-11** | **5115018** |
| ID8-12 | 418 |
| ID8-13 | 1433454 |
| ID8-14 | 818 |
| **ID8-15** | **2558656** |
